# Novel Intrinsic Mechanisms of Active Drug Extrusion at the Blood-Brain Barrier: Potential Targets for Enhancing Drug Delivery to the Brain?

**DOI:** 10.3390/pharmaceutics12100966

**Published:** 2020-10-14

**Authors:** Wolfgang Löscher, Birthe Gericke

**Affiliations:** 1 Department of Pharmacology, Toxicology, and Pharmacy, University of Veterinary Medicine, Bünteweg 17, D-30559 Hannover, Germany; birthe.gericke@tiho-hannover.de; 2 Center for Systems Neuroscience, D-30559 Hannover, Germany

**Keywords:** P-glycoprotein, ABCB1, protein trafficking, lysosomal sequestration, extracellular vesicles, neutrophils

## Abstract

The blood-brain barrier (BBB) limits the pharmacotherapy of several brain disorders. In addition to the structural and metabolic characteristics of the BBB, the ATP-driven, drug efflux transporter P-glycoprotein (Pgp) is a selective gatekeeper of the BBB; thus, it is a primary hindrance to drug delivery into the brain. Here, we review the complex regulation of Pgp expression and functional activity at the BBB with an emphasis on recent studies from our laboratory. In addition to traditional processes such as transcriptional regulation and posttranscriptional or posttranslational modification of Pgp expression and functionality, novel mechanisms such as intra- and intercellular Pgp trafficking and intracellular Pgp-mediated lysosomal sequestration in BBB endothelial cells with subsequent disposal by blood neutrophils are discussed. These intrinsic mechanisms of active drug extrusion at the BBB are potential therapeutic targets that could be used to modulate P-glycoprotein activity in the treatment of brain diseases and enhance drug delivery to the brain.

## 1. Introduction

The blood-brain barrier (BBB) provides a natural defense against toxic or infective agents circulating in the blood but also restricts the brain penetration of most drugs, thus forming a bottleneck in drug development for brain diseases [1,2]. Tight junctions between brain capillary endothelial cells (BCECs) significantly reduce the permeation of small hydrophilic solutes through the intercellular cleft (paracellular pathway), thus forming a “physical barrier” [3]. The tight junctions ultimately determine the barrier properties of the BBB, but adherens junctions, which mediate the initial adhesion between endothelial cells, play a modulatory role [4]. Thus, most drugs must use predominantly transcellular pathways to reach the brain parenchyma, which is only possible in case of favorable physicochemical properties or active transport by membrane transporters of the solute carrier (SLC) family [1]. Small, lipophilic, and uncharged compounds, such as anesthetic agents, can penetrate relatively freely through the BBB by passive diffusion to reach their targets in the brain. However, many of such compounds are subject to active efflux by promiscuous ATP-binding cassette (ABC) transporters such as P-glycoprotein (Pgp; MDR1; ABCB1), breast cancer resistance protein (BCRP; ABCG2) or multidrug resistance proteins (MRPs; ABCCs) that are located at the apical, blood-facing membrane of BCECs and pump drugs back into the blood before they reach the brain parenchyma [1]. As a consequence, several therapeutically important drug categories, including antiepileptic drugs (AEDs; now typically termed anti-seizure drugs [ASDs]), antidepressant drugs, anticancer drugs, or certain anti-infectious agents are restricted in their brain penetration by efflux transporters such as Pgp, which may result in the drug resistance of brain diseases [1,5,6,7]. In theory, this problem may be easily resolved by the coadministration of Pgp inhibitors. Indeed, several Pgp inhibitors or modulators have been investigated in clinical trials in patients with brain cancer or pharmacoresistant epilepsy, with limited success [2,6,8]. Several alternative strategies are actively being pursued, such as the modification of existing drugs, the development of new drugs, or the combination of novel drug delivery agents to evade Pgp-dependent efflux [9,10]. For any of these strategies, it is important to understand the transport mechanisms and regulation of ABC transporters such as Pgp at the BBB. Furthermore, the brain disease itself may alter the expression and functionality of efflux transporters at the BBB, as shown for certain types of difficult-to-treat epilepsy [2], which needs to be dealt with when developing new therapies.

**Figure 1:**
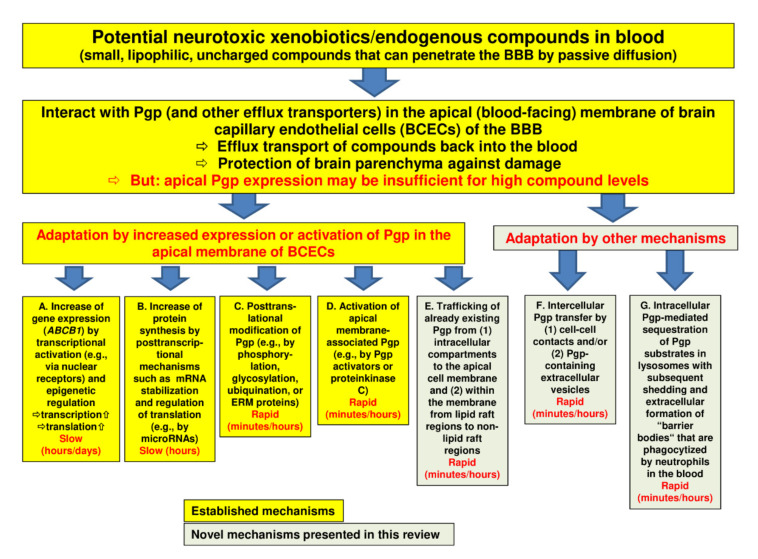
Regulation of P-glycoprotein (Pgp) expression and functionality at the blood-brain barrier. The figure illustrates a number of processes by which Pgp may adapt to high blood levels of xenobiotics with the aim to protect the brain parenchyma from intoxication. The yellow boxes (designated by (**A**–**C**)) show well-known mechanisms of Pgp adaptation (see Section 2), whereas the greenish boxes (**E**–**G**) illustrate the novel mechanisms that we characterized in brain capillary endothelial cells in recent years (see Section 3). Note that the mechanisms described in boxes (**A**,**B**,**F**) will increase the Pgp content of the cell, whereas the mechanisms described in boxes (**C**,**D**,**E**) and (**G**) will not. Although Pgp is the best characterized drug efflux transporter at the blood-brain barrier (BBB), other transporters such as breast cancer resistance protein (BCRP) and multidrug resistance proteins (MRPs) are thought to support Pgp in its role to protect the brain (see Section 2.6). Indeed, the role of these other transporters is often underestimated, particularly in older studies.

In order to protect the brain from intoxication, Pgp (and other efflux transporters) at the BBB should be capable of rapidly adapting their expression or activity to high levels of a potentially harmful xenobiotic in the blood (Figure 1). However, most of the adaptation processes that have been previously described are relatively slow. Thus, we hypothesized that there should be intrinsic mechanisms of active drug extrusion at the BBB that respond more rapidly to high-level drug exposure. This hypothesis was examined in a series of studies, which will be described in Section 3 of this review, after shortly discussing more conventional mechanisms of Pgp regulation at the BBB.

## 2. Regulation of Pgp at the BBB: Conventional Mechanisms

In the absence of ABC transporters such as Pgp, potentially harmful xenobiotics that are small, uncharged, and lipophilic would freely penetrate through the BBB and intoxicate the brain. Pgp, a 170 kDa plasma membrane protein that is the best-characterized drug efflux transporter at the human BBB, restricts the brain entry of a variety of structurally unrelated compounds and thus prevents their accumulation within the brain with the goal to protect the parenchyma against damage [1,11]. In order to fulfill this goal, Pgp expression and functionality in the BBB need to be tightly regulated in response to xenobiotics [8,12,13,14,15]. Indeed, a variety of mechanisms can alter Pgp transporter expression and transport function at the BBB. These mechanisms, which are illustrated in Figure 1A–D, are reviewed in the following.

### 2.1. Transcriptional Activation of Pgp by Ligand-Activated Nuclear Receptors

A number of ligand-activated nuclear receptors function as sensors for endogenous metabolites, therapeutic drugs, and environmental toxicants [16]. These “orphan” nuclear receptors, including the pregnane X receptor (PXR; also known as the steroid and xenobiotic sensing nuclear receptor {SXR} or NR1I2 {nuclear receptor subfamily 1, group I, member 2}), the farnesoid X receptor (FXR; or NR1H4 {nuclear receptor subfamily 1, group H, member 4}), and the constitutive androstane receptor (CAR; also known as NR1I3 {nuclear receptor subfamily 1, group I, member 3}), are key transcriptional regulators that autonomously coordinate the body’s response to potentially harmful compounds by affecting their absorption, distribution, metabolism, and excretion in the intestine, liver, and kidney [16]. Ligand-activated nuclear receptors are also expressed at the BBB, where they regulate enzymes and transporters in the brain capillary endothelium [13].

Nuclear receptors are transcription factors that are activated by ligand binding, which initiates the transcription of their target genes. PXR, FXR, and CAR are activated by endogenous compounds such as steroids, bile acids, as well as by exogenous xenobiotics, including a large number of therapeutic drugs [13]. At the BBB, nuclear receptor activation increases the protein expression and transport activity of Pgp, BCRP, and MRPs such as MRP2, providing a mechanism by which xenobiotics can signal to tighten the BBB to themselves and to multiple foreign chemicals [12,17]. However, because this process involves enhanced transcription of the respective transporter genes, the path from the activation of nuclear receptors to increased transporter protein expression will be slow (Figure 1A). For instance, in vitro, the PXR ligand rifampicin increased Pgp protein expression in brain capillaries of hPXR transgenic mice after 6 h of exposure, whereas in vivo, this was seen after a 3-day treatment with rifampicin [18]. The PXR-driven upregulation of Pgp at the BBB was associated with reduced analgesic activity of the Pgp substrate methadone, which was the first demonstration of the ability of BBB PXR to alter the efficacy of a drug that acts on the central nervous system (CNS) [18].

A variety of structurally diverse drugs can induce *ABCB1* gene expression in the BBB and other tissues by transcriptional activation (often mediated by nuclear receptors), including anti-seizure drugs (ASDs) such as phenobarbital, phenytoin, and carbamazepine, viral protease inhibitors such as saquinavir and nelfinavir, antibiotics such as rifampicin, glucocorticoids such as dexamethasone and prednisolone, analgesics such as morphine, and cytostatic drugs such as doxorubicin and vinblastine [19,20,21]. However, in in vitro studies using BCECs from different species, including humans, we found marked differences in the efficacy of known Pgp inducers to increase Pgp activity [22,23]. Such species differences are also known for ligand-activated nuclear receptors [24] and should be kept in mind when translating data across species.

### 2.2. Transcriptional Activation of Pgp by Epigenetic Regulation

In addition to nuclear receptors, epigenetic regulation may lead to the transcriptional activation of Pgp (Figure 1A). For instance, epigenetic modifications of the *ABCB1* proximal and far upstream promoters by either the demethylation of DNA or acetylation of histone H3 play a pivotal role in inducing *ABCB1* expression [25]. Histone acetylation is an epigenetic modification that can regulate gene expression by changing the accessibility of the genome to transcriptional regulators and transcriptional machinery [26]. Previous studies have suggested that the pharmacological inhibition of histone deacetylases (HDACs) modulates the expression and function of Pgp and BCRP as a result of enhanced histone acetylation [27]. In an in vitro model of the human BBB, the human BCEC line hCMEC/D3 (Human Cerebral Microvascular Endothelial Cell Line clonal population D3), enhanced histone acetylation in response to HDAC inhibitors increased *ABCB1* mRNA and Pgp protein levels and Pgp transport activity [28]. This effect of HDAC inhibitors involved facilitated binding of the aryl hydrocarbon receptor (AhR) at the *ABCB1* promoter. The AhR, a member of the bHLH-PAS family of DNA-binding proteins, is a cytoplasmic protein that upon ligand binding translocates to the nucleus, where it heterodimerizes with AhR nuclear translocator (ARNT) and binds to specific enhancer sequences adjacent to the promoter element of certain genes [29]. AhR is expressed at the BBB where it can upregulate mRNA and the protein expression of ABC transporters such as Pgp [15,30].

The epigenetic regulation of Pgp expression and activity in the BBB is a slow process (Figure 1A); the functional consequences of HDAC inhibition typically develop slowly during treatment with HDAC inhibitors. Interestingly, the ASD valproate (VPA) acts as an HDAC inhibitor [31] and was shown to increase Pgp expression and functionality in hCMEC/D3 cells [23,28]. Furthermore, the inhibition of HDACs by VPA and trichostatin A (TSA) enhanced intercellular Pgp transfer in hCMEC/D3 cells [32], which will be further discussed in Section 3.2.

### 2.3. Other Mechanisms of Transcriptional Activation of Pgp

**Figure 2:**
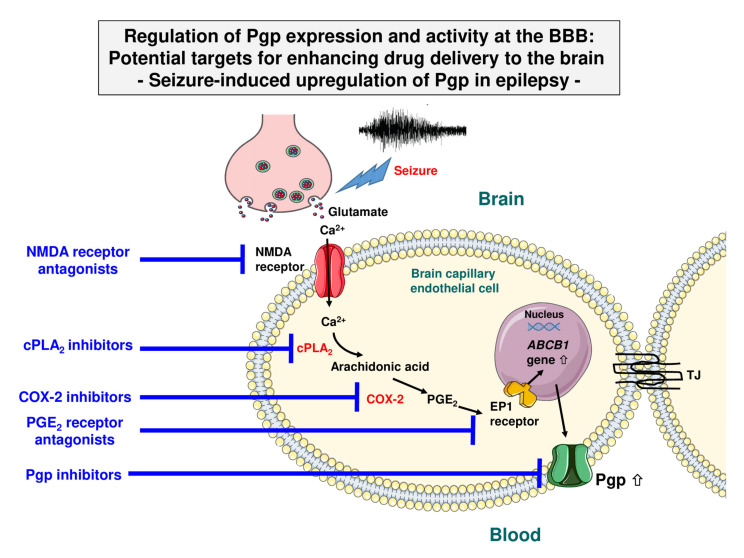
Signaling pathway in brain capillaries mediating the seizure-induced overexpression of Pgp. Seizure-induced release of glutamate signals through the N-methyl-D-aspartate (NMDA) receptor, which leads to activation of cytosolic phospholipase A2 (cPLA_2_). In turn, this leads to the release of arachidonic acid, which cyclooxygenase 2 (COX-2) converts to PGE_2_, which, via the PGE_2_ (EP1) receptor, leads to the transcriptional activation of *ABCB1* (*MDR1*) and thus increased Pgp expression and transport activity levels at the BBB. The downstream event of EP1 receptors, which drives transcriptional activation of the Pgp encoding gene, is not clear, but the transcription factor NF-κB (nuclear factor κB) has been proposed to act as a master regulator of ATP-binding cassette (ABC) transporter expression in brain capillaries [35]. Note that the different cell and vesicle types are not drawn to scale. Modified from Potschka [36] and Hartz et al. [33].

In addition to various drugs, endogenous compounds can induce Pgp expression (Figure 2). An interesting example is glutamate, an excitatory neurotransmitter released during epileptic seizures, resulting in Pgp and BCRP overexpression at the BBB [13]. This effect of glutamate is likely mediated by cytosolic phospholipase A2 (cPLA_2_) [33]. The overexpression of efflux transporters at the BBB is thought to be involved in ASD resistance in epilepsy [7]. Thus, based on the data of Hartz et al. [33], preventing transporter overexpression through cPLA_2_ inhibition could potentially serve as a novel therapeutic strategy to increase ASD brain uptake and reduce seizure burden. As shown in Figure 2, the proposed signaling pathway by which glutamate increases Pgp and BCRP expression and activity in brain capillaries starts with the seizure-induced release of neuronal glutamate, which signals through the glutamate N-methyl-D-aspartate (NMDA) receptor, leading to the activation of cPLA_2_ in capillaries [33]. In turn, this leads to the release of arachidonic acid, which cyclooxygenase 2 (COX-2) converts to prostaglandin E_2_ (PGE_2_), which, via PGE_2_ (EP1) receptors, leads to the transcriptional activation of Pgp and BCRP at the BBB [12,15,33,34]. The downstream events of EP1 receptors, which drive the transcriptional activation of the Pgp and BCRP encoding genes, still need to be identified, but the transcription factor NF-κB has been proposed to act as a master regulator of ABC transporter expression in brain capillaries [35].

Several previous in vivo studies in rodent models have shown that this signaling cascade can be pharmacologically interrupted (Figure 2), e.g., by NMDA receptor antagonists, COX-2 inhibitors, and PGE_2_-receptor antagonists, which prevent the seizure/glutamate-induced increase of transporter expression and activity [37,38,39,40,41]. Furthermore, ASD resistance can be counteracted by such pharmacological interruption in rodent models [42], most likely by increasing the brain penetration of ASDs such as phenytoin or phenobarbital into the brain [41]. Using the same rat model of ASD resistant epilepsy, we previously reported a similar beneficial effect on ASD resistance for the Pgp inhibitor tariquidar [43].

Since NMDA receptor antagonists (e.g., memantine) and COX-2 inhibitors (e.g., celecoxib) are clinically used, this interesting approach of treating ASD resistance could be easily translated to the clinical arena. However, this would necessitate demonstrating that the glutamate-induced upregulation of Pgp and BCRP described above also occurs in humans. In a series of experiments in a human BCEC line (hCMEC/D3), we never observed increased Pgp expression or transport activity by exposure to glutamate (100 µM for 0.5 to 2 h; A. Noack and W. Löscher, unpublished data), but we found that glutamate modulated the Pgp association with lipid rafts in the cell membrane (see Section 3.1). However, using freshly prepared human brain capillaries, glutamate (100 µM for 0.5 h) was found to upregulate Pgp-mediated efflux transport, which was blocked by NMDA receptor and COX-2 antagonists [44]. Similarly, glutamate was reported to upregulate MRP2 in human and porcine brain capillaries, which was inhibited by NMDA receptor and COX-2 antagonists [45]. In apparent contrast, Salvamoser et al. [46] reported the glutamate-mediated downregulation of BCRP in primary cultured porcine and human BCECs, whereas Hartz et al. [33] found that glutamate increased BCRP protein and activity levels in isolated rat brain capillaries.

Recently, Hartz et al. [47] performed a study that combined in vivo and ex vivo approaches to assess Pgp protein expression and transport activity levels in models of seizures and epilepsy in the rat, as well as in brain capillaries from patients with ASD-resistant epilepsy. The data showed that exposing brain capillaries to glutamate ex vivo and that acute seizures and chronic epilepsy (with spontaneous recurrent seizures) in vivo upregulate both Pgp protein expression and transport activity to a similar extent, which is in line with the transporter hypothesis of ASD-resistant epilepsy [7]. As illustrated in Figure 2, enhanced understanding of how Pgp activity increases in epileptogenic tissue provides potential targets for enhancing drug delivery to the brain in epilepsy.

In addition to inducing Pgp expression in endothelial cells of the BBB, glutamate and transforming growth factor beta (TGF-β) are involved in BBB disruption, which is a hallmark of most types of epilepsy, leading to the extravasation of albumin and subsequent functional brain alterations [2]. A combination of losartan (an angiotensin II type 1 receptor antagonist that blocks brain TGF-β signaling) and clinically available NMDA receptor antagonists such as memantine may be advantageous in preventing BBB disruption in a wide variety of brain diseases, including epilepsy, stroke, Alzheimer’s disease, and traumatic brain injury [48].

### 2.4. Posttranscriptional Mechanisms in Pgp Adaptation to Xenobiotics

Recent studies have demonstrated that microRNAs (miRNAs) contribute to the maintenance of the BBB and thereby mediate the homeostasis of the CNS [49]. miRNAs are small non-coding RNA molecules of 20–24 nucleotides that function in RNA silencing and the posttranscriptional regulation of gene expression [50]. Several miRNAs mediate the posttranscriptional control of Pgp [50]. miRNAs leading to an upregulation of Pgp expression (Figure 1B) include miR-27a, miR-138, miR-296, and miR-451 [50]. Other mRNAs, including miR-298, mediate the downregulation of Pgp [50]. Recently, it was shown that miR-298 reverses multidrug resistance to ASDs by downregulating Pgp expression in the BBB, using human BCECs [51].

Another posttranscriptional mechanism in Pgp modulation is alteration in *ABCB1* mRNA stabilization (Figure 1B). For instance, acute exposure to the Pgp substrate ivermectin led to the overexpression of functional Pgp in mouse liver cells through increased stability of mRNA in the cell [52]. Pgp in the BBB prevents almost exclusively the penetration of ivermectin into the brain [11]. mRNA stabilization could play a role in this respect. However, similar to the other transcriptional and posttranscriptional mechanisms illustrated in Figure 1A,B, this process will be too slow to allow the rapid adaptation of Pgp to high drug levels in the blood.

### 2.5. Posttranslational Mechanisms in Pgp Adaptation to Xenobiotics

Posttranslational modifications (Figure 1C) have been shown to modulate the functional expression of Pgp and other ABC transporters via a wide range of molecular mechanisms [53]. These modifications commonly occur through the addition of a functional group (e.g., phosphorylation), a small protein (e.g., ubiquitination), sugar chains (e.g., glycosylation), or lipids (e.g., palmitoylation) on solvent accessible amino acid residues. Such modifications are critical for the transporters’ structure, function, and regulation within the confines of the lipid environment [53]. For instance, the phosphorylation of Pgp has been shown to regulate Pgp-mediated drug resistance, while glycosylation was reported to influence the stability of surface-expressed Pgp [53]. Another pathway of posttranslational regulation of Pgp localization and function involves the scaffold proteins ezrin, radixin, and moesin (ERM proteins) [54]. For different cell types, including the BBB, it has been shown that Pgp is glycosylated posttranslationally and subsequently trafficked to and inserted into the plasma membrane, where activated ERM proteins link with Pgp to maintain its membrane localization and functional activity [54]. This is relevant for Pgp adaptation at the BBB to xenobiotics as suggested by a study of Kobori et al. [55], who reported that an increase in moesin expression might contribute to an increased expression of Pgp at the BBB, leading to the development of morphine analgesic tolerance. In hCMEC/D3 cells, moesin knockdown caused a large decrease in P-gp and BCRP efflux activity [56].

### 2.6. Activation of Membrane-Associated Pgp

In addition to transcriptional, posttranscriptional, and traditional posttranslational mechanisms that increase Pgp expression in the apical plasma membrane of BCECs, a new class of compounds has recently been identified (and designated as Pgp activators) that immediately increase Pgp activity without increasing its protein expression [20,57,58]. Such compounds act by binding to a specific ligand-binding site, inducing a conformational change in Pgp that stimulates the efflux of a substrate bound on another ligand-binding site. Therefore, this activation mechanism promotes Pgp transport function without interfering with protein expression levels, making it a more rapid process than Pgp induction (Figure 1D). These findings are consistent with a functional Pgp model containing at least two positively cooperative sites for drug binding and transport [20]. Pgp activators include drugs such as prazosin, progesterone, and nonsteroidal anti-inflammatory drugs (NSAIDs) such as mefenamic acid, sulindac, naproxen, and meloxicam [20].

In addition to drugs acting as Pgp activators, protein kinase C (PKC) modulates Pgp activity, but the direction of the modulation (activation or inhibition) depends on the PKC isoenzyme [12,15,59,60]. The concept of Pgp activators provides interesting therapeutic strategies because promoting the Pgp-mediated efflux of deleterious xenobiotics will result in a significant reduction in their intracellular levels and, consequently, in a significant reduction of their toxicity [20].

### 2.7. Compensatory Function of BCRP and Other Drug Efflux Transporters at the BBB

Apart from the adaptive mechanisms illustrated in Figure 1, other ABC efflux transporters at the BBB may help Pgp prevent high blood levels of xenobiotics from intoxicating the brain parenchyma. BCRP is of particular importance in this respect [6]. Using quantitative targeted absolute proteomics of the human and mouse BBB transporters, determined by means of a liquid chromatography-tandem mass spectrometric quantification method, Uchida et al. [61] reported that BCRP is the most abundantly expressed drug transporter in human brain microvessels (BCRP expression levels were about 30% higher than Pgp expression levels), while Pgp expression was higher than BCRP expression in mouse brain microvessels. However, Uchida et al. [61] measured the BCRP monomer, and since BCRP is a half-transporter that needs to homodimerize to be active, the BCRP quantity reported by Uchida et al. [61] needs to be divided by two in order to estimate the amount of functionally active BCRP. Furthermore, Uchida et al. [61] did not differentiate between BCRP in the luminal membrane (that would presumably contribute to efflux transport) vs. BCRP in subapical vesicles (that would not contribute to efflux transport). Consequently, the relative functional activity of BCRP vs. Pgp at the human or rodent BBB is currently not known. However, irrespective of the relative expression of BCRP vs. Pgp at the BBB, it is clear that BCRP and Pgp have an overlapping and synergistic role in restricting the entrance of xenobiotics into the brain [6,62]. Indeed, a systematic series of experiments in mouse models with the deletion of Pgp, BCRP, or both suggest a remarkable compensatory function for the two transporters [6]. PET studies in humans provided evidence for a functional interplay between Pgp and BCRP at the human BBB and suggested that both efflux transporters need to be inhibited to achieve substantial increases in the brain distribution of dual Pgp/BCRP substrates [63]. Furthermore, MRPs expressed at the BBB may contribute to the restriction of drug penetration into the brain as well [64,65].

In addition to the expression of Pgp, BCRP, and MRPs in BBB endothelial cells, these transporters are also expressed in foot processes of astrocytes that form a complex network surrounding the brain capillaries and contribute to the induction and maintenance of the barrier properties [3]. Transporter expression by perivascular astrocyte endfeet may act as a “second line of defense” if the primary barrier is breached, dysfunctional, or overloaded.

## 3. Regulation of Pgp at the BBB: Novel Mechanisms

As discussed above and shown in Figure 1, transcriptional and posttranscriptional mechanisms of Pgp modulation are too slow to allow the rapid adaptation of transporter activity to increased demand. The activation of membrane-associated Pgp by Pgp activators is a more rapid process, but this is not a likely adaptation mechanism in the scenario illustrated in Figure 1. Thus, we thought about alternative mechanisms that would rapidly increase Pgp at the apical membrane and investigated such mechanisms in in vitro models of the BBB.

### 3.1. Intracellular Pgp Trafficking

In most cells, Pgp is primarily localized on the plasma membrane for its efflux function; however, it is also localized in intracellular compartments [66]. These intracellular localizations link to synthesis (endoplasmic reticulum), modification (Golgi), traffic/recycling (Golgi and endosomes), and degradation (lysosome and proteasome) sites for Pgp. Pgp cycles between endosomal compartments and the cell surface in a microtubular-actin dependent manner [66]. Modulating Pgp trafficking from intracellular compartments to the cell surface alters posttranscriptional Pgp expression and may be an effective and rapid way for the cell to respond to potentially toxic compounds by inserting functional efflux transporter into the membrane (Figure 1E).

**Figure 3:**
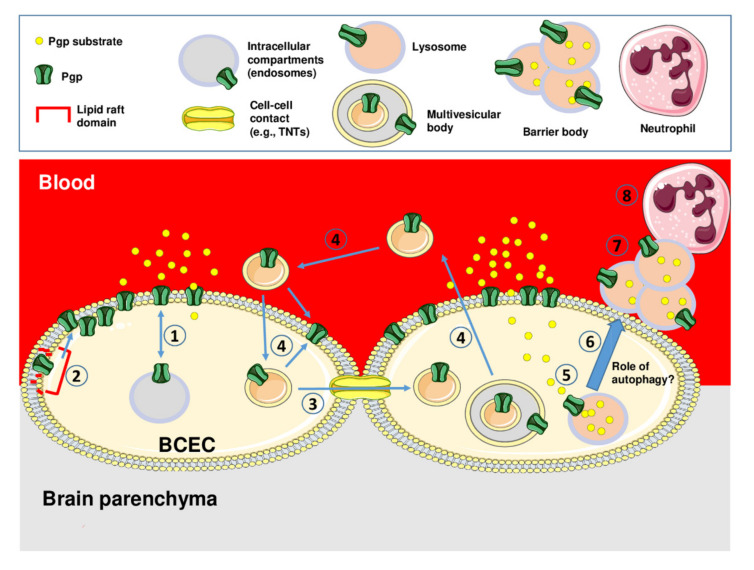
Schematic illustration of intra- and intercellular trafficking of Pgp and Pgp-mediated lysosomal drug sequestration and disposal in brain capillary endothelial cells that form the BBB. Intracellular trafficking of Pgp from intracellular endosomal compartments to the apical membrane is illustrated by (1), while the trafficking of Pgp within the membrane from lipid raft regions to non-lipid raft regions is illustrated by (2). Intercellular Pgp trafficking may occur via cell-to-cell contacts, e.g., tunneling nanotubes as indicated by (3), or via the transfer of Pgp-containing extracellular vesicles (e.g., exosomes released from multivesicular bodies [MVBs]) as indicated by (4). The intracellular Pgp-mediated sequestration of Pgp substrates in lysosomes is illustrated by (5). Subsequently, lysosomes containing the Pgp substrate leave the cell, e.g., by fusing with autophagosomes to autolysosomes (6), and form aciniform apical aggregates (“barrier bodies”; (7)) that are phagocytized by neutrophil granulocytes (8). Note that the different cell and vesicle types are not drawn to scale. See text for details.

Newly synthesized Pgp can be delivered to the cell surface in different ways [66]. The constitutive pathway involves Pgp incorporation into transport vesicles that move directly to the plasma membrane along the cytoskeleton [67]. The second pathway is via the intracellular endosomal system in which Pgp-containing endosomal compartments (endocytic plasma membrane-derived vesicles) form an intracellular pool, from which Pgp can be delivered to the cell surface [66], as illustrated in Figure 3 (1). Indeed, the endolysosomal system is substantially involved in protein trafficking, which is a process that is dependent on the proper function of membrane-integrated proteins and ion channels in endolysosomal vesicles [68].

Pgp trafficking and recycling is performed with a group of proteins, which are commonly known as Ras-associated binding (Rab) proteins [69]. Rab proteins are part of the Ras superfamily of small GTPases known to regulate most vesicular transport events by regulating vesicle docking and fusion [70,71]. There are more than 60 Rab proteins in mammalian cells, and most of them are localized in the subcellular membrane compartment, where they control the intracellular trafficking routes of proteins and lipids [72]. These Rab proteins are often associated with Pgp. The involvement of Rab6 is observed during the trafficking of Pgp from Golgi apparatus to the plasma membrane, whereas Rab11 and Rab13 are involved in the trafficking of Pgp from Golgi apparatus to the recycling endosome, and Rab11a has been shown to be involved in the trafficking of Pgp to the apical membrane in polarized cells [66,69]. In BBB endothelial cells, the co-fractionation of Rab5 and Rab11a with Pgp indicated that endosomal/lysosomal pathways are potentially involved in Pgp trafficking in vivo [73]. Furthermore, Rab4 was reported to modulate the surface expression of Pgp in cancer cells [74], and we found that Pgp and Rab4 are associated with the human and rat BCEC lines hCMEC/D3 and RBE4 [75].

The intracellular trafficking of Pgp has been demonstrated for different cell types, particularly liver and cancer cells [66,76,77,78,79]. Until recently, little was known about the trafficking mechanisms of Pgp and their regulation in BCECs that form the BBB. Studying Pgp transport activity in porcine BCECs and freshly isolated brain capillaries from pigs, Ott et al. [60] reported that the plant flavonoid quercetin (a St. John’s Wort constituent) increased Pgp transport activity (as indicated by calcein-acetoxymethyl (AM) uptake), which was blocked by brefeldin A, an inhibitor of vesicular trafficking between the endoplasmatic reticulum and the Golgi apparatus. Based on this observation, Ott et al. [60] suggested that the increased Pgp activity caused by quercetin was a result of trafficking and membrane insertion of subapical vesicles that contain transporter protein, but no direct evidence for this hypothesis was provided. Likewise, Hartz et al. [80], using isolated rat brain capillaries, suggested that PKC may influence Pgp trafficking in BCECs, stimulating retrieval from the plasma membrane into a vesicular compartment, as has been demonstrated for Pgp in hepatocytes [67], but, again, no direct evidence was provided. A study by McCaffrey et al. [81], using rat cerebral microvessels, demonstrated an induction of Pgp trafficking at the BBB by peripheral inflammatory pain in rats and indicated that this stimulus promotes a dynamic redistribution between membrane domains of Pgp and caveolin-1, which is a key scaffolding/trafficking protein associated with caveolar microdomains also known as lipid rafts. Membrane (lipid) rafts and caveolae, a subset of rafts, are cellular microdomains that concentrate plasma membrane proteins and lipids such as cholesterol involved in the regulation of cell function [82]. Interestingly, glutamate changes lipid raft composition via the activation of PKC [83], which may add to the effects of glutamate on Pgp discussed in Section 2.3.

Several years ago, at the annual *Barrier and Transporter Meeting* in Bad Herrenalb (Germany), Andreas Reichel presented data showing that—following drug exposure—subapical Pgp-containing vesicles fusion within 1–3 min with the luminal membrane of BCECs, which was confirmed by Björn Bauer in Gert Fricker’s lab. However, to our knowledge, these data were not published [84].

We investigated the intracellular trafficking of Pgp in human hCMEC/D3 cells, which we stably transduced with a doxycycline-inducible *MDR1*-EGFP (Enhanced Green Fluorescent Protein) fusion plasmid [85]. hCMEC/D3 cells express endogenous Pgp (and other efflux transporters), but the expression of Pgp is lower in hCMEC/D3 cells than in freshly prepared human brain microvessels, although Pgp remains functional as shown by uptake assays with Pgp substrates [86,87]. In the presence of doxycycline, *MDR1*-EGFP-transduced hCMEC/D3 cells exhibit a 15-fold increase in Pgp protein expression, which is associated with an increased efflux of the Pgp substrate rhodamine 123 [85]. *MDR1*-GFP constructs have been previously used to study intracellular localization and trafficking of Pgp in other cell types [77,88,89,90,91,92,93], but to our knowledge, a tetracycline regulatory system to control the induction of Pgp expression has not been previously described. As shown in our study [85], Pgp expression can be effectively controlled in this system, providing an ideal environment for studying cellular trafficking of the protein and interactions with a variety of intracellular targets.

To examine drug-induced trafficking of Pgp, we used the chemotherapeutic agent mitomycin C (MMC), which has previously been shown to increase membrane-associated Pgp by inducing Pgp trafficking in rat hepatoma (H4IIE) and *MDR1*-GFP transduced canine kidney (MDCK) cells [90]. In contrast to various other chemotherapeutic agents, MMC is not a Pgp substrate, or at best a poor Pgp substrate, and it does not induce *MDR1* mRNA [94]; thus, Pgp trafficking is possibly the only way by which the cell can protect itself against this toxic compound.

In our experiments, using confocal fluorescence microscopy of single hCMEC/D3-*MDR1*-EGFP cells, we demonstrated that Pgp redistribution from intracellular pools to the cell surface occurred within 2 h of MMC exposure. Pgp-EGFP exhibited a punctuate pattern at the cell surface compatible with concentrated regions of the fusion protein in membrane microdomains, i.e., lipid rafts, which was confirmed by Western blot analysis of biotinylated cell surface proteins in Lubrol-resistant membranes. MMC exposure also increased the functionality of Pgp as assessed in three functional efflux assays with Pgp substrates (rhodamine 123, eFluxx-ID Gold, and calcein-AM), which could be blocked by the Pgp inhibitor tariquidar. However, the increase in Pgp functionality occurred with some delay after the increased Pgp expression and coincided with the release of Pgp from the Lubrol-resistant membrane complexes. Disrupting lipid rafts by depleting the membrane of cholesterol by methyl-β-cyclodextrin increased the functionality of Pgp. Furthermore, glutamate modulated the Pgp association with lipid rafts in endothelial cells [75].

At the BBB, Pgp is mainly localized in caveolin-1/flotillin-2 positive caveolar microdomains (known as lipid rafts), and its activity can be modulated by interaction with caveolin-1 [79,82]. This localization in microdomains most likely explains the clustered formation of Pgp-EGFP observed in our study and a study by Huber et al. [95] in hCMEC/D3 cells, using the super-resolution fluorescence microscopy method by spectral precision distance microscopy/spectral position determination microscopy (SPDM) to investigate the spatial distribution of Pgp in the luminal plasma membrane of brain capillary endothelial cells.

In addition to Pgp in intracellular compartments, such as the endoplasmic reticulum, the Golgi apparatus, various endosomes, lysosomes, and proteasomes, which are important in the synthesis, posttranslational modification, traffic/recycling, and degradation of Pgp, the nuclear envelope is a place for intracellular Pgp localization [96]. In chemotherapy-resistant cancer cells, the nuclear localization of Pgp is responsible for protection of the nucleus from chemotherapeutics such as doxorubicin [97]. However, Pgp can also traffic from the nuclear envelope to the luminal surface of cells [98]. More recently, Schaefer et al. [99] reported that chronic morphine exposure potentiates pain-induced Pgp trafficking from nuclear reservoirs to the luminal surface of freshly isolated cortical rat brain microvessels. Since morphine is a Pgp substrate, this observation suggested that Pgp trafficking contributes to the decreased morphine analgesic effects in morphine-tolerant rats experiencing an acute pain stimulus.

In summary, our 2014 paper [85] presented the first direct evidence of drug-induced Pgp trafficking at the human BBB (Figure 1E) and indicated that at least in part, Pgp has to be released from lipid rafts to gain its full functionality (process (2) in Figure 3). This offers several targets for reducing Pgp activity at the BBB and thereby enhancing drug delivery to the brain, which will be discussed in Section 4.1.

### 3.2. Intercellular Pgp Transfer

The intercellular transfer of proteins is an integral part of communication between cells, involving mechanisms such as “tunneling nanotubes” (TNTs) bridging neighboring cells for the release and uptake of protein-containing extracellular vesicles [100]. TNTs, which contain a straight, continuous actin rod enclosed in a lipid bilayer, are long thin membranous tubes that interconnect cells (Figure 3, (3)), representing a relatively novel route of cell-to-cell communication and the spreading of pathogens [100,101]. TNTs, which were first described by Rustom et al. in 2004 [102], are reported to exchange mitochondrial and lysosomal organelles as well as intracellular vesicles, RNA, membrane proteins and cellular cargo between donor and recipient cells [100,101]. TNTs have been found in many prokaryotic and eukaryotic cells, including neurons, myeloid cells, T cells, kidney cells, cardiac myocytes, and endothelial cells, and they are becoming a promising therapeutic target in cancer and neurodegenerative diseases [103]. In addition to TNTs, gap junction channels enable the direct cell-cell transfer of metabolic, biochemical, and electric signals [104]. However, it is currently not known which of these or other types of cell-cell junctions may mediate the intercellular transfer of Pgp, as illustrated in Figure 3.

**Figure 4:**
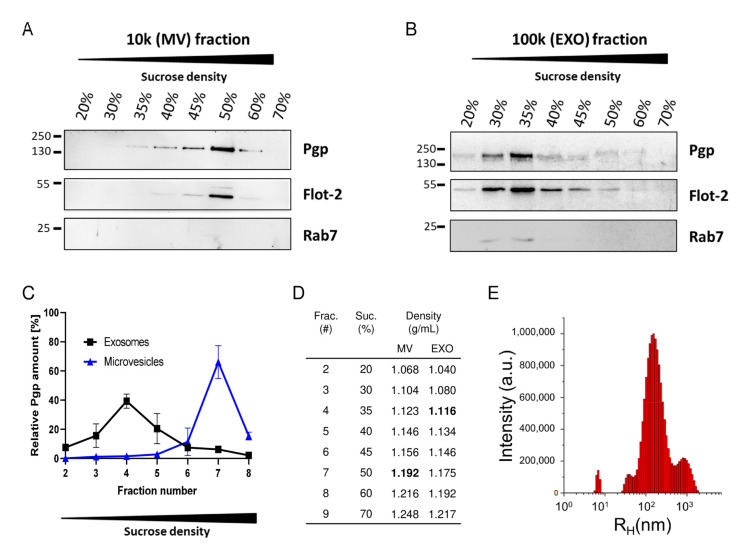
Fractionation of extracellular vesicle subtypes released by doxorubicin-treated hCMEC/D3 (Human Cerebral Microvascular Endothelial Cell Line clonal population D3) cells and analysis of Pgp content. Conditioned medium from doxorubicin-treated hCMEC/D3-cocultures was subjected to differential centrifugation and density gradient centrifugation followed by Western blot analysis of the (**A**) 10,000× *g* microvesicle (MV) and the (**B**) 100,000× *g* exosome (EXO) isolates after sucrose gradient fractionation (bottom-up flotation). The fractions were probed for Pgp, the lipid raft marker flotillin-2 (Flot-2), and the small GTPase Rab7. (**C**) Relative Pgp protein distribution among sucrose fractions of increasing density collected from gradients loaded with EXO (black line) or MV (blue line) isolates (*n* = 2). In accordance with the smaller size of EXOs in comparison to the Pgp-containing MV exosome, isolates mainly distribute to a fraction with low sucrose concentration, whereas Pgp-containing MVs float at a higher sucrose concentration. (**D**) Table presenting sucrose (suc.) concentration (in %) and corresponding densities of the different fractions (frac.) after gradient centrifugation. (**E**) Size distribution of the EXO isolate was determined by dynamic light scattering. The size of particles is given as hydrodynamic radius (R_H_) in nm, and relative abundance is given as arbitrary units (a.u.). The size distribution histogram of the EXO preparation shows a prominent species at ≈200 nm (R_H_). Data are from unpublished experiments of Birthe Gericke and Felix Osten.

A second process that allows the intercellular transfer of proteins is mediated by cell-derived extracellular vesicles (Figure 3, (4)). Extracellular vesicles are broadly divided into three main groups according to their biogenesis, size, and molecular composition: (i) exosomes (≈30–100 nm in diameter), (ii) microvesicles (also termed ectosomes, shedded vesicles, or microparticles; ≈100–1000 nm in diameter), and (iii) apoptotic bodies (≈1000–5000 nm in diameter) [105]. Exosomes originate from the exocytosis of multivesicular bodies, i.e., a type of late endosome containing internal vesicles formed following the inward budding of the outer endosomal membrane. The typically larger microvesicles originate from direct outward budding of the cellular plasma membrane. Apoptotic bodies are released by the membrane blebbing of dying cells and may contain DNA and histones. Extracellular vesicles can be released from nearly all cell types, constitutively, and/or upon induction [106]. Rab GTPases such as Rab5, Rab7, Rab11, Rab27, and Rab35 are involved in both the biogenesis and secretion of extracellular vesicles such as exosomes [107].

In 2005, Levchenko et al. [108] reported that the intercellular transfer of Pgp mediates acquired multidrug resistance (MDR) in tumor cells, most likely by the cell-cell contact-mediated transfer of Pgp. This report added another dimension to the ways that cells can acquire a particular cell surface protein-mediated phenotype, such as multidrug resistance [100]. Since then, numerous studies have confirmed the finding of Levchenko et al. [108], showing that intercellular Pgp transfer occurs in different cancer cell lines, and exploring mechanisms involved in this transfer [69,109,110,111,112].

We investigated if intercellular Pgp transfer as reported for cancer cells is also a physiological defense mechanism of BCECs that form the BBB [32]. Using cocultures of hCMEC/D3 wild-type and hCMEC/D3-*MDR1*-EGFP cells that allow visualizing the fate of the Pgp-EGFP fusion protein, we found that the Pgp-EGFP fusion protein was transferred from donor to recipient cells by cell-to-cell contact (likely via TNTs) and Pgp-EGFP enriched vesicles (likely exosomes), which were exocytosed by donor cells and endocytosed by adherent recipient cells as illustrated in Figure 1F and Figure 3 (4). Flow cytometry experiments with the Pgp substrate eFluxx-ID Gold demonstrated that transferred Pgp is functional in the recipient cells. Exposure of the donor cells with inhibitors of HDACs (TSA and VPA) resulted in an enhanced intercellular Pgp transfer. This could explain the increased Pgp expression and resistance to chemotherapeutics that have been reported for the exposure of cancer cells to HDAC inhibitors [113].

Non-genetic transfer of a resistance phenotype and its regulation by HDACs is a novel mechanism of altering BBB functionality. This mechanism may have important implications for understanding drug-induced alterations in Pgp expression and activity at the BBB. The clinical significance of this mechanism will depend on whether the transfer of Pgp at critical levels occurs in vivo. We plan to study this question by positron emission tomography (PET) and ^11^C-labeled Pgp substrates such as (*R*)-[^11^C]-verapamil [114], following treatment with and without HDAC inhibitors. As shown previously, the advantage of this strategy is that it can be rapidly translated from experimental animals to humans [115,116].

To further characterize the nature of Pgp-loaded extracellular vesicles (EVs) involved in intercellular Pgp transfer, we differentiated microvesicles (ectosomes) and exosomes released by cocultures of hCMED/D3-*MDR1*-EGFP and hCMEC/D3 wild-type (WT) cells following treatment with doxorubicin (Figure 4). The different types of extracellular vesicles, which differ in size (see above), were separated by differential centrifugation followed by discontinuous sucrose step-gradient fractionation. Both microvesicle (MV) and exosome (EXO) fractions contained Pgp, and—according to size differences of the EV subtypes—there was a difference in the sucrose density of the Pgp-enriched fractions between EXO (1.116 g/mL) and MV (1.192 g/mL) isolates after bottom-up flotation in the sucrose gradient. Furthermore, both microvesicle and exosome isolates contained the lipid raft marker flotillin-2, which is consistent with the literature of flotillin-positive extracellular vesicles [117], whereas Rab7, which has been implicated in multivesicular body (MVB) formation (from which exosomes originate) and fusion with the plasma membrane [118]), was determined only in exosomal fractions. These data substantiate that Pgp-containing exosomes/extracellular vesicles were involved in the intercellular Pgp transfer described by Noack et al. [32].

### 3.3. Intracellular Sequestration of Pgp Substrates in Lysosomes and Subsequent Disposal

Pgp is synthesized in the endoplasmic reticulum and, as discussed above, trafficked along the secretory pathway through the Golgi apparatus to the cell surface, but it is also localized to endosomes and lysosomes [119]. Pgp localization in endosomes has been suggested to serve as an intracellular reservoir before Pgp moves to the cell surface, while—until recently—lysosomes were thought to be mainly responsible for Pgp degradation [119,120]. Cells utilize two major pathways for intracellular protein degradation: the endosomal-lysosomal system and the non-lysosomal ubiquitin-proteasome system. The lysosomal degradation system is the primary pathway responsible for the fate of cell surface Pgp, which has a half-life of approximately 30 h in human cancer cells [120].

In the BBB, lysosomal degradation has been suggested to be a mechanism for restricting the access of xenobiotics to the brain [121,122]. Indeed, lysosomal degradation represents a big challenge for brain drug delivery because the majority of pharmaceutical agents end up in lysosomes instead of being transcytosed across the BBB [121,122].

Recent evidence indicates that the importance of the lysosome in cell metabolism and organism physiology goes far beyond the simple degradation of proteins and extracellular particles by a number of proteases and lipases [123]. For instance, it has previously been demonstrated that most of the lipophilic and weak basic chemotherapeutic drugs, such as doxorubicin, can accumulate in acidic lysosomes (lumen pH ≤ 5), especially in drug-resistant cancer cells, which can be reversed by the alkalinization of lysosomes [124,125]. Therefore, the sequestration of chemotherapy drugs in lysosomes, followed by either degradation or transport to the cell surface and extrusion into the external space, is widely considered to be a bona fide mechanism of resistance to weakly basic chemotherapy drugs in cancer cells. Indeed, the reducing and acidic environment of lysosomes causes the protonation of certain weak basic cytotoxic agents, such as doxorubicin, trapping these molecules within the organelle, such that they are unable to reach their cellular targets, e.g., the nucleus [125,126]. In addition to cancer cells, lysosomal trapping of cationic drugs has also been demonstrated in retinal capillary endothelial cells that form the blood-retinal barrier [127,128].

In addition to passive drug sequestration (or trapping) of hydrophobic weak base chemotherapeutics in lysosomes, other mechanisms of lysosome-mediated drug resistance of cancer cells have also been reported. These include active lysosomal drug sequestration mediated by ABC transporters such as Pgp, BCRP, or MRP1 [129,130,131,132,133,134]. This active lysosomal drug sequestration is thought to be enabled by the topological inversion of Pgp and other ABC transporters via endocytosis (lysosomes are formed via endocytosis of the plasma membrane), resulting in the transporter actively pumping agents into the lysosome [125,126]. The lysosomal sequestration of doxorubicin in cancer cells was reported to be dependent on Pgp activity, as an inhibition of Pgp drug transport by Pgp inhibitors (elacridar, valspodar) or siRNA led to a loss of lysosomal sequestration of doxorubicin [132].

Pgp primarily transports neutral and cationic hydrophobic compounds, while MRP substrates are primarily anionic drugs and their glucuronidated, sulfated, and glutathione-conjugated metabolites [8]. The substrate profile for BCRP includes sulfoconjugated organic anions as well as hydrophobic and amphiphilic compounds. Thus, among other factors, the physicochemical properties of a drug determine which transporter mediates its lysosomal sequestration, although there is considerable overlap in the substrate specificity of ABC transporters. In addition to ABC transporters such as Pgp, MRP1, or BCRP, several SLC transporters are expressed in BCECs and contribute to the active transport of drugs at the BBB [8,135,136,137].

Another interesting aspect of the lysosomal sequestration of drugs such as doxorubicin is that lysosomal drug accumulation activates the transcription factor EB (TFEB), the master regulator of lysosomal biogenesis [68]. Thus, lysosomal drug accumulation induces lysosomal biogenesis and thus lysosomal sequestration capacity [138]. Furthermore, TFEB acts as a transcription factor for several proteins that are essential for autophagy, which is a complex process promoting cell survival during stress conditions and a driving factor for the chemoresistance of cancer cells [68].

Most of our knowledge about the functions of the endolysosomal system stems from studies on polarized epithelial or cancer cells. The emergence of advanced microscopy techniques has begun to provide more detailed information on this system in BCECs, including the characterization of the endolysosomal system in primary cultured porcine BCECs [139] and hCMEC/D3 cells in vitro [140] and mouse BCECs in vivo [141]. Furthermore, the role of lysosomal functions in BCECs under pathological conditions has become an emerging area of research [122,142].

Prompted by Pgp-mediated lysosomal trapping in cancer cells, we recently studied if the lysosomal sequestration of Pgp substrates, including doxorubicin, also occurs in BCECs that form the BBB [143]. As in our previous studies on intra- and intercellular Pgp trafficking, *MDR1*-EGFP transduced hCMEC/D3 cells were used as a model. In addition to this widely used cell line, primary cultured porcine BCECs, which naturally exhibit high levels of Pgp [144], were used to exclude that any findings in the immortal hCMEC/D3-*MDR1*-EGFP- or WT cell lines were secondary to the genetic manipulations of these cells [145].

**Figure 5:**
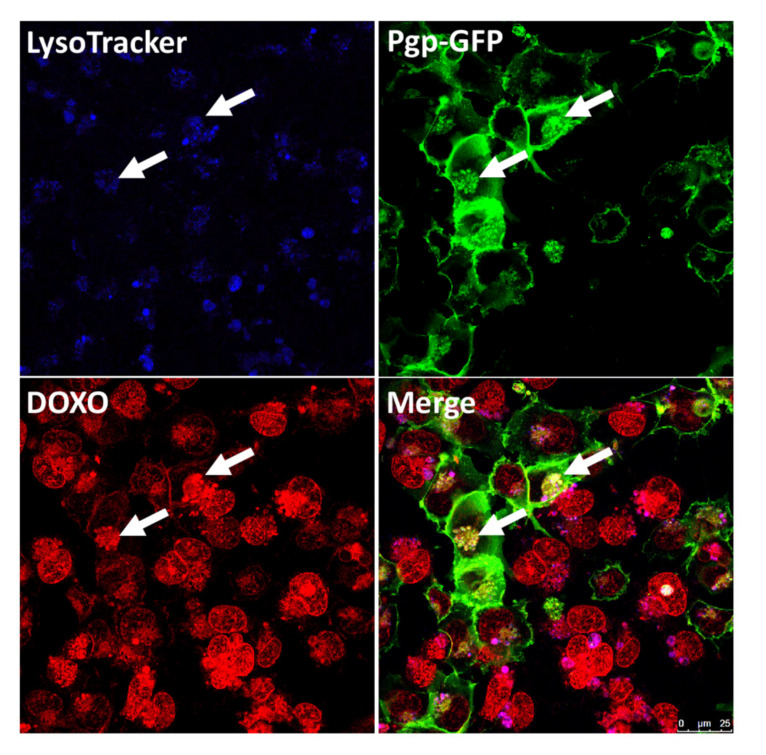
Sequestration of doxorubicin (DOXO) into Pgp-EGFP positive endolysosomal vesicles in hCMEC/D3 cocultures. After confluency, hCMEC/D3 cocultures (equal amounts of WT and *MDR1*-EGFP transduced cells) were incubated with the blue fluorescent dye LysoTracker Blue DND-22 (75 nm, 1 h, 37 °C) to label acidic endolysosomal compartments, followed by treatment with the Pgp substrate DOXO (10 µM, 30 min, 37 °C), which is visible in red by its autofluorescent character. Cells were subsequently analyzed by live-cell imaging and confocal microscopy. Arrows indicate the DOXO sequestration into endolysosomal Pgp-EGFP (green staining) enriched compartments within the brain capillary endothelial cells. Unpublished figure from B. Gericke.

Our experiments demonstrated that the endolysosomal trapping of Pgp substrates occurs in human and porcine BCECs (Figure 1G, Figure 3 (5), Figure 5) [143]. Unexpectedly, following endolysosomal drug sequestration, we observed subsequent shedding of these Pgp substrate-sequestering vesicular structures (Figure 3 (6)). These structures stayed attached to the apical side of the BCEC plasma membrane and formed aciniform aggregates, which we termed “barrier bodies” (Figure 3 (7)). Scanning electron microscopy substantiated the budding of vesicles from the apical membrane of BCECs after treatment with Pgp substrates and the accumulation of the vesicles in aciniform aggregates at the apical cell surface. To our knowledge, such membrane-attached Pgp/substrate sequestering structures have not been described for BCECs or any other Pgp containing cell type.

**Figure 6:**
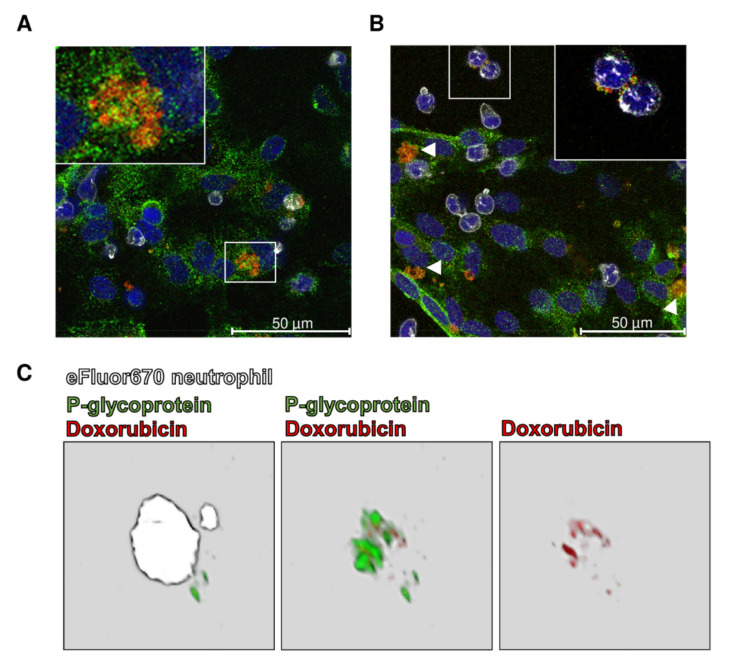
Barrier-body formation and ingestion by porcine neutrophils after treatment of primary porcine brain capillary endothelial cells (pBCECs) with doxorubicin. (**A**) pBCECs were isolated from porcine brain and treated with doxorubicin (10 µM, 37 °C, 30 min; depicted in red) 5 days after culturing. Freshly isolated porcine neutrophils were labeled with the fluorescent dye eFluor670 (depicted in white) and incubated with the pBCEC culture for 10 min, 37 °C. Cells were fixed with aceton-methanol, indirectly stained for P-glycoprotein (Pgp, depicted in green), and analyzed by confocal fluorescence microscopy. Nuclei were counterstained with 4′,6-diamidino-2-phenylindole (DAPI; depicted in blue). Doxorubicin localizes in Pgp-positive perinuclear vesicular structures (inset in (**A**)) identified as endolysosomal vesicles (see Ref. 140). The white frame outlines the section magnified (inset). (**B**) The doxorubicin-containing vesicles were released by the pBCECs and form aggregates (barrier bodies; arrowheads) at the apical surface of the cells. The inset in (B) highlights the ingestion of barrier bodies by neutrophils. (**C**) 3D reconstruction of an eFluor670 stained neutrophil that ingested a barrier body after incubation with doxorubicin-treated pBCECs. The 3D reconstruction was performed with Leica Application Suite (LASX, version 1.9.0.13747). The first micrograph in (**C**) shows a merge of the three channels eFluor670 (white), Pgp (green), and doxorubicin (red). The barrier body, ingested by the neutrophil (white), consists of a Pgp-containing membrane that encloses doxorubicin, which becomes visible when the eFluor670 channel is masked out (micrographs 2 and 3 in (**C**)). Unpublished figure from B. Gericke.

The localization of barrier-body aggregates at the plasma membrane border of adjacent endothelial cells and the presence of both the WT marker eFluor670 and Pgp-EGFP in the barrier bodies indicated that each barrier-body aggregate (which was exhibited by ≈10% of the endothelial cells) was formed by vesicles from more than one endothelial cell. This suggests that at least 20% of the cells in the cocultures of hCMEC/D3 *MDR1*-EGFP and hCMEC/D3 WT cells contributed to barrier-body formation [143]. The same processes of lysosomal drug sequestration, barrier-body formation and phagocytosis by neutrophils were also observed in primary cultured porcine BCECs [143] (Figure 6). Thus, barrier bodies are not a non-physiological phenomenon (or artifact) that occurr only in *MDR1*-EGFP transduced hCMEC/D3 cells, but they also occurred in WT hCMEC/D3 cells that received Pgp-EGFP from donor cells and in primary cultures of pBCECs. The exact origin and mechanisms involved in barrier body formation have to be clarified in more detail in future studies.

The extracellular localization of these structures and their attachment to the apical cell membrane of the BCECs led us to hypothesize that the formation and shedding of the barrier bodies may be an effective way for the cell to dispose of cytotoxic compounds via phagocytic blood cells and, thus, provide a second-line defense mechanism against cytotoxic drugs [143]. Indeed, when *MDR1*-EGFP-transduced hCMEC/D3 cells were cocultured with human primary blood-derived neutrophils, the blood cells extended pseudopods directed toward the BCEC plasma membrane, presumably hunting for potential target antigens, followed by phagocytosis of the barrier bodies (Figure 3 (8)). In the search for signals that attracted the neutrophils, we found that BCEC exposure to doxorubicin significantly increased the release of interleukin (IL)-8, which is known to be chemotactic for neutrophils. Additional experiments showed that the barrier body formation was blocked by the inhibition of vesicular trafficking and significantly reduced by the inhibition of Pgp [143].

Why has this surprising process of shedding of drug-sequestering endolysosomal vesicles, which stay attached to the apical side of the plasma membrane and form aggregates that are phagocytosed by neutrophils, not been previously reported? At least three methodological prerequisites were crucial for our observations. First, the conditional doxycycline-inducible *MDR1*-EGFP-expressing hCMEC/D3 cells allowed the visualization of Pgp within and outside the BCECs. Second, we first observed this process by using the Pgp substrate eFluxx-ID Gold (EFIG), which is a xanthene-based small-molecule dye coupled to acetoxymethyl (AM) ester (EFIG-AM) to allow cell permeability; EFIG-AM has advantages compared with more commonly used Pgp substrates because the hydrophobic, nonfluorescent EFIG-AM readily penetrates the cell membrane, where it is hydrolyzed by intracellular esterases to a hydrophilic fluorescent metabolite (EFIG) that cannot enter intracellular vesicles by passive diffusion [146]. Thus, unless EFIG is actively transported out of the cell or sequestered in intracellular compartments by active transport, the esterase cleaved dye is trapped inside the cell. Therefore, this feature favored the detection of Pgp-mediated lysosomal sequestration in BCECs. In this respect, EFIG differs from the more commonly used Pgp substrates, such as weak basic chemotherapeutic agents (e.g., doxorubicin), which can be sequestered in lysosomes in the absence of multidrug transporters such as Pgp by pH partitioning (see above). Third, to our knowledge, interactions between cocultures of BCECs and neutrophils have not been investigated previously for Pgp-mediated drug disposal. After we characterized this novel process of drug disposal with EFIG-AM in hCMEC/D3 *MDR1*-EGFP cells, we substantiated our findings with another Pgp substrate (doxorubicin) and primary cultured porcine BCECs (Figure 6) [143].

Several open questions remain to be addressed. To our knowledge, shedding or outward budding of lysosomes has not been described previously, although lysosomes are able to fuse with the plasma membrane and release their content into the extracellular space via an exocytic pathway, which has been shown to be involved in resistance to chemotherapeutic agents [123]. One potential pathway for the cargo delivery observed in our study would be the fusion of lysosomes with autophagosomes (or amphisomes), leading to autolysosomes [147,148]. Autophagy is a lysosomal degradation pathway for cytoplasmic components; increasing evidence suggests that both autophagy and lysosomal drug sequestration play a role in acquired resistance to doxorubicin in cancer cells [149,150]. The autolysosomes or the vesicles of endolysosomal origin are released by the subsequent outward budding or protrusion of the plasma membrane, which might explain the enclosure of barrier-body vesicles by a Pgp-containing plasma membrane as observed in our experiments [143]. Indeed, the electron microscopic examination of hCMEC/D3 cells after exposure to doxorubicin (10 µM, 30 min) revealed the formation of autophagic vacuoles (indicating the induction of autophagy) and increased internalization (endocytosis) of cell surface Pgp (unpublished data).

In addition to Pgp, other ABC transporters such as MRP1 and BCRP can be expressed by lysosomes and mediate lysosomal drug sequestration [128,131]. Both doxorubicin and EFIG are substrates of Pgp, as well as BCRP and MRPs [65,146], which may add to the lysosomal drug sequestration observed in our BCEC experiments, although the colocalization of Pgp-EGFP and EFIG, as well as doxorubicin in both lysosomal vesicles and barrier bodies, indicates that the sequestration was mainly mediated by Pgp [143].

Finally, some potential caveats need to be considered. First, the in vivo relevance of lysosomal drug sequestration and barrier body formation, as observed in our study, remains unknown at this stage. The demonstration of these processes in vivo will be a challenging task that will be addressed in future experiments. One strategy that can be used for such experiments is PET with radiolabeled Pgp substrates, as described by Kannan et al. [151]. Furthermore, the process of active Pgp-mediated drug sequestration in lysosomes as reported by Yamagishi et al. [132] and other groups in cancer cells and by us in BCECs [143] can be debated because of a lack of clear, unequivocal evidence that Pgp is expressed in the lysosomal membrane. The presence of Pgp in lysosomes has been visually demonstrated by confocal microscopy analysis and the co-localization of fluorescent anti-fluorescein isothiocyanate (FITC)-Pgp antibody as well as lysosomal markers, such as LAMP2 and LysoTracker [152]. However, confocal microscopy does not allow discriminating between Pgp in the lysosomal lumen or membrane. The limiting lysosome membrane comprises more than 200 integral membrane proteins, including a proton-importing V-type ATPase that maintains the acidic pH of the lumen and a set of highly-glycosylated lysosome-associated membrane proteins (LAMPs) that protect the membrane from degradation by the lysosomal hydrolases [153]. In a proteomic analysis of lysosomal membranes of liver lysosomes, nine ABC transporters were identified, but Pgp was not among them [154]. We currently investigate this issue in liver and BCEC lysosomes by electron microscopy with immunogold antibody labeling of LAMP-2 and Pgp.

## 4. Novel Intrinsic Mechanisms of Active Drug Extrusion at the BBB: Potential Targets for Enhancing Drug Delivery to the Brain?

Apart from conventional strategies, such as the blockade of drug efflux at the BBB by the systemic administration of Pgp inhibitors, which has the disadvantage that Pgp activity is suppressed throughout the body, more specific strategies interfere with the mechanisms involved in the regulation of the transporter. One example—Pgp activation in epilepsy—is illustrated in Figure 2 and has been discussed in Section 2.3. As proof-of-principle that interfering with the endogenous processes involved in the upregulation of Pgp at the BBB increases brain uptake of Pgp substrates in vivo, van Vliet et al. [41] reported that the inhibition of COX-2 in epileptic rats counteracts enhanced Pgp expression and increases brain levels of the ASD (and Pgp substrate) phenytoin. Similar strategies to interfere with the endogenous processes involved in the regulation of Pgp activity (and the activity of other BBB efflux transporters) exist for brain cancer, ischemia, neuroinflammation, Alzheimer’s disease, and others [8,12,35,54,155,156].

In this section, we will address the pharmacological manipulation of the novel mechanisms of Pgp adaptation described in Section 3 and illustrated in Figure 3. Although these mechanisms provide potential novel targets for enhancing drug delivery to the brain, their preclinical evaluation and potential translation to the clinic is in its infancy.

### 4.1. Targeting Intracellular Pgp Trafficking

**Figure 7:**
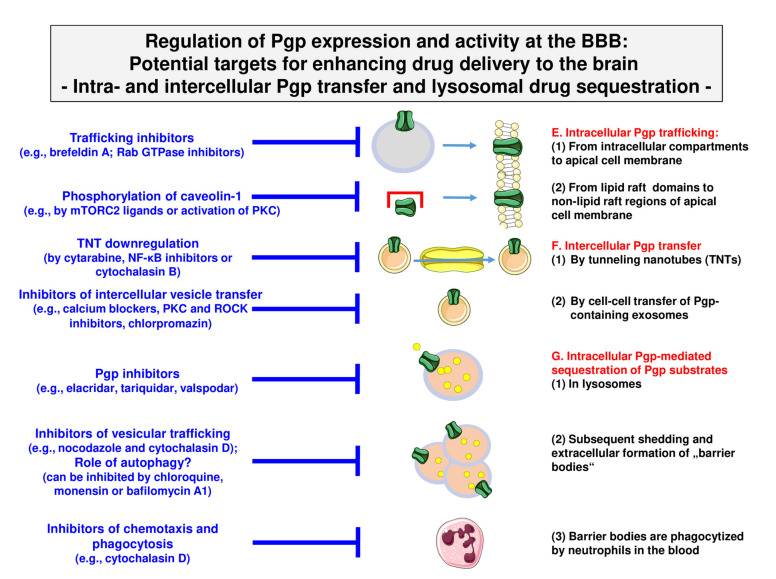
Pharmacological inhibition of the processes involved in intra- and intercellular Pgp trafficking and Pgp-mediated lysosomal drug sequestration. Note that the different cell and vesicle types are not drawn to scale. See Figure 3 and text for details.

As outlined in Section 3.1, intracellular Pgp trafficking from intracellular reservoirs to the cell surface is an effective and rapid way for the cell to respond to potentially toxic compounds by functional membrane insertion of the efflux transporter. Thus, this mechanism will also contribute to restricting the brain entry of therapeutically used Pgp substrates. As shown in Figure 7, intracellular Pgp trafficking in BCECs can be blocked by the antiviral, antibiotic brefeldin A, which is an inhibitor of vesicular protein trafficking between the endoplasmatic reticulum and the Golgi apparatus [60].

Another strategy to alter Pgp trafficking is targeting proteins that co-localize with Pgp during trafficking, such as Rab GTPases (see Section 3.1). Of particular interest are Rab6 and Rab11a, which are involved in the trafficking of Pgp from subcellular pools to the apical membrane [69]. Targeting Rabs has been proposed as a novel therapeutic strategy for cancer therapy [157] but may also be interesting for increasing drug delivery across the BBB. However, the development of selective Rab GTPase inhibitors has proven challenging, with no direct inhibitors identified for clinical trials thus far. Although specific Rab GTPase inhibitors are desirable for drug development, pan-GTPase inhibitors such as CID1067700 can be used to study the role of Rab GTPases in Pgp trafficking [158]. Furthermore, indirect strategies for targeting Rab GTPases became available recently, such as modulating Rab-membrane association and inhibiting Rab-effector interactions [157].

As described in Section 3.1, in the cell membrane, Pgp is partially localized in membrane microdomains (lipid rafts or caveolae), where it colocalizes with the scaffolding/trafficking protein caveolin-1. We have shown that disrupting lipid rafts by depleting the membrane of cholesterol with methyl-β-cyclodextrin increased the functionality of Pgp in hCMEC/D3 cells [85]. This was unexpected, because cholesterol decreases membrane fluidity, which should favor the functionality of Pgp as reported for tumor cell lines and fibroblasts [82,159,160]. Thus, these data indicate that the acute depletion of cholesterol impacts Pgp-mediated drug transport in a cell-type- or cell-line-specific manner. Similar to our observations with methyl-β-cyclodextrin in BCECs, the downregulation of caveolin-1 by siRNA increased Pgp functionality in the rat BCEC line RBE4 [161]. Inversely, manipulations that increased Pgp colocalization with caveolin-1, such as the stimulation of caveolin-1 phosphorylation on tyrosine-14, decreased Pgp functionality in RBE4 cells [161]. Thus, the binding of Pgp to caveolin-1 (Pgp has a binding motif in its N-terminus for caveolin-1) negatively regulates Pgp function. Interestingly, the phosphorylation of caveolin-1 on tyrosine-14 and caveolae formation are regulated by mechanistic target of rapamycin (mTOR) complex 2 (mTORC2), which is a key effector of the mTOR pathway [162]. Furthermore, the activation of PKC, which is known to modulate caveolar function, increases the phosphorylation of caveolin-1 [163].

### 4.2. Targeting Intercellular Pgp Transfer

As described in Section 3.2, we found in BCECs that Pgp is transferred from Pgp-overexpressing donor cells to recipient wild-type cells by cell-to-cell contact (most likely via TNTs) and Pgp-EGFP enriched exosomes, which were exocytosed by donor cells and endocytosed by adherent recipient cells, as illustrated in Figure 3 (3) and (4). Intercellular Pgp transfer is known to contribute to multidrug resistance in cancer cells [69,100,109] and likely also plays a role in the rapid adaptation of the BBB to high blood levels of xenobiotics, as illustrated in Figure 1F. Thus, the mechanisms underlying intercellular Pgp transfer provide interesting targets for enhancing drug delivery to the brain.

As shown in Figure 7, cell-to-cell transfer via TNTs can be inhibited by TNT downregulation by compounds such as cytarabine, NF-κB inhibitors, and cytochalasin B or latrunculin B [164,165]. The production and release of extracellular vesicles can be inhibited in various cell types by diverse compounds, including calcium channel blockers, ROCK (rho-associated protein kinase) inhibitors, calpain inhibitors, PKC inhibitors, methyl-β-cyclodextrin, chlorpromazine, and the vitamin B5 derivative pantethine [111,166]. Furthermore, Rab GTPases play a role in vesicle trafficking [69,71,166] and thus provide targets for interfering with this process [157]. Consistent with the many successful examples of in vitro drug-induced suppression of extracellular vesicle production and/or release, there are now numerous exciting clinical examples where this has also been achieved in vivo [166]. Achieving a therapeutic inhibition of extracellular vesicle production is the focus of much research and several clinical trials. It remains to be evaluated whether such strategies can be used to enhance drug delivery to the brain.

### 4.3. Targeting Intracellular Sequestration of Pgp Substrates in Lysosomes and Subsequent Disposal

As described in Section 3.3, we recently reported a novel second-line defense mechanism in BCECs—that is, Pgp-mediated intracellular lysosomal drug trapping, followed by the shedding of lysosomal Pgp/substrate complexes at the apical membrane of human and porcine BBB endothelial cells and the subsequent phagocytosis of these “barrier bodies” by neutrophils [143]. As illustrated in Figure 3 and Figure 4, the individual processes involved in this novel mechanism of drug disposal at the BBB provide several targets for interference. First, as shown by several groups (for a review, see Stefan et al. [152]), Pgp-mediated lysosomal drug sequestration can be inhibited by Pgp inhibitors. Furthermore, depending on the respective drug, other ABC transporters such as BCRP and MRP1 seem to be involved in Pgp-mediated lysosomal drug sequestration, which can be inhibited pharmacologically [131]. In cancer cells, the inhibition of lysosomal drug sequestration by ABC transport inhibitors has been shown to reduce or prevent drug resistance, whereas, depending on the chemotherapeutic drug being studied, the disruption of lysosomal pH alone was not always effective in this regard [125,152]. Interestingly, when we exposed cocultures of hCMEC/D3-*MDR1*-EGFP and hCMEC/D3 WT cells with the Pgp/BCRP inhibitor elacridar, we observed that barrier body formation was reduced by 50% when added at its *IC*_50_ for Pgp (0.2 μM), thus substantiating the involvement of Pgp in this process [143].

Barrier body formation was completely suppressed by inhibitors of vesicular trafficking, i.e., nocodazole and cytochalasin D [143]. Nocodazole interferes with the polymerization of microtubules, whereas cytochalasin D is a cell-permeable and potent inhibitor of actin polymerization, and both actin filaments and microtubules are involved in different membrane traffic pathways [167].

As discussed in Section 3.3, one likely mechanism underlying our barrier body observation is the formation of Pgp substrate-containing autolysosomes that are released from the cell by outward budding or protrusion of the plasma membrane [143]. Autophagy can be inhibited by lysosomotropic agents such as chloroquine, hydroxychloroquine, and monensin, which are taken up selectively into lysosomes and block the fusion of lysosomes with autophagosomes, or the macrolide antibiotic bafilomycin A1 [168]. These drugs have been shown to reduce the resistance of cancer cells to chemotherapy. We currently study whether this approach reduces barrier body formation in BCECs, as indicated in Figure 7.

Apart from leukocyte transendothelial migration in neuroinflammatory processes [169,170], the potential interaction of blood cells such as neutrophils with BBB regulatory mechanisms is not well understood. Thus, our recent observation [143] that neutrophils scan the plasma membrane of BCECs and phagocytize barrier bodies is highly interesting, and they also offer targets for pharmacological manipulation of this process (Figure 7). Several drugs have been described to inhibit neutrophil chemotaxis and phagocytosis, including membrane-penetrating sulfhydryl reagents such as cytochalasin D and tricyclic antidepressants [171,172]. Furthermore, our observation that BCECs release IL-8, which is a chemotactic for neutrophils, upon exposure to Pgp substrates such as doxorubicin indicates that the interaction between neutrophils and BCECs can be blocked by drugs that inhibit IL release. This issue is currently under investigation.

## 5. Experimental In Vivo Strategies for Translation of In Vitro Findings on Pgp Regulation at the BBB

As yet, the novel intrinsic mechanisms of Pgp-mediated active drug extrusion at the BBB described here have been characterized only in BBB in vitro models. As shown by our previous studies, *MDR1*-EGFP-transduced hCMEC/D3 cells can be used as a tool for studying principal mechanisms of drug uptake, intracellular sequestration, trafficking, and extrusion, which then have to be confirmed by primary BCEC cultures and, ultimately, in in vivo BBB models. As described above, one strategy that can be used for such in vivo experiments is brain PET with radiolabeled Pgp substrates, as for instance described by Kannan et al. [151] for lysosomal drug trapping. We have used PET extensively to study the role of Pgp vs. BCRP in drug efflux at the mouse, rat, and human BBB [173,174,175,176,177,178,179,180,181,182,183,184,185,186] and to investigate the effect of epilepsy on Pgp expression in brain regions [115].

**Figure 8:**
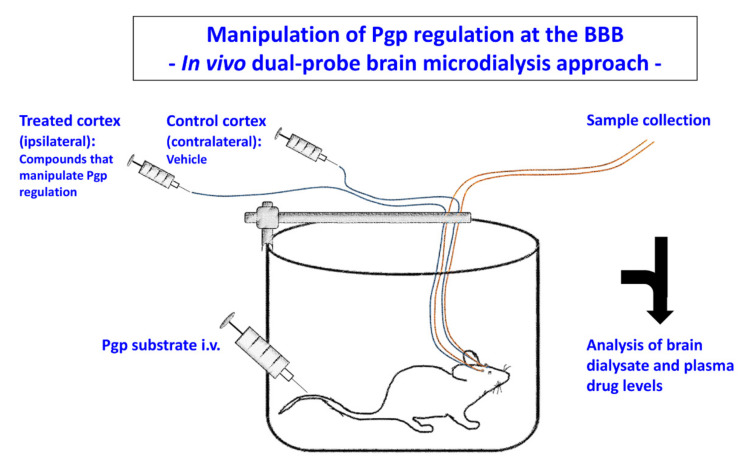
Dual-probe intracerebral microdialysis in studies on Pgp-mediated restriction of drug penetration across the BBB. The experimental protocol used by us for this purpose is similar to the protocol described by Burgio et al. [195] for studying the involvement of Pgp in the control of brain distribution of the chemotherapeutic agent etoposide. In short, rats are implanted with two microdialysis probes in the left and right motor cortices. One probe is infused with an aqueous Ringer’s solution containing a compound that interferes with Pgp regulation at the BBB (e.g., a Pgp inhibitor) and the other probe is infused with Ringer’s solution and the vehicle used for dissolving the compound. Then, a Pgp substrate is injected systemically (intraperitoneally or intravenously), and plasma and dialysate concentrations are repeatedly determined over the course of an experiment in conscious, freely moving rats. Since only one cortex is treated with a compound, the vehicle-treated cortex served as a control site in each individual rat. Redrawn and modified from Löscher and Potschka [187].

However, PET has a limited spatial resolution and does not allow differentiating between functionally relevant unbound drug levels and bound drug in the brain parenchyma. We have previously used another approach, which is illustrated in Figure 8 and utilizes dual-probe brain microdialysis. In this approach, rats are bilaterally implanted with microdialysis probes in the cerebral cortex, and brain extracellular fluid and blood are repeatedly sampled during an experiment in freely moving animals [64,187]. Following the systemic administration of a Pgp substrate, a compound that manipulates a mechanism involved in Pgp regulation is administered via the microdialysis probe in one hemisphere, whereas vehicle is administered in the other (control) hemisphere. Comparison of extracellular brain drug levels between the treated and control hemispheres allows determining the effect of manipulation of Pgp regulation. We have previously used this experimental design for determining the effects of Pgp and MRP inhibitors on brain levels of ASDs [188,189,190,191,192], but in principle, all the compounds described in Figure 7 can be evaluated using this approach. A similar approach has been used by Sziráki et al. [193,194] in rats and mice for in vitro and in vivo correlation studies on Pgp substrates and inhibitors.

Another strategy that we recently used to investigate BBB transport mechanisms involved in the brain uptake and efflux of the loop diuretic bumetanide is the intracerebroventricular (i.c.v.) administration of transport inhibitors after systemic drug administration [196]. Again, i.c.v. probes are bilaterally implanted in rats (or mice), allowing the comparison of brain drug levels in a treated hemisphere with a vehicle-treated control hemisphere.

To study the role of barrier body formation by BCECs and subsequent phagocytosis by blood neutrophils, we plan to use an experimental in vivo approach for endothelial-derived extracellular vesicles previously described by Paul et al. [197]. In a series of experiments, these authors provided evidence that during inflammation, the tight junction protein claudin-5 is transferred from BBB endothelial cells via the release of extracellular vesicles to circulating leukocytes in mice in vivo. EGFP-labeled claudin-5 was used in these experiments. In our experiments, we plan to treat rats or mice with subtoxic doses of doxorubicin to induce lysosomal sequestration and barrier body formation at the BBB (which we will examine in brain capillaries ex vivo), followed by the sorting of LAMP-2-positive barrier body-containing neutrophils in the blood. The functional role of barrier body formation will be studied in rodent models of ASD-resistant epilepsy, as previously used for characterizing the role of drug efflux transporters such as Pgp for drug resistance in epilepsy [7].

## 6. Conclusions

The vast majority of previous studies that tried to enhance drug delivery to the brain by interfering with Pgp at the BBB used drugs that directly inhibit Pgp. In the ensuing 30 years, three distinct generations of Pgp inhibitors have been developed [6,198]. First-generation inhibitors, including verapamil, quinidine, amiodarone, and cyclosporine A, were not selective, not potent, or were toxic. The second-generation agents valspodar (PSC833) and dexverapamil were more potent but interfered with drug metabolism. The third generation of inhibitors, including dofequidar, zosuquidar, tariquidar, elacridar, and biricodar, were developed specifically as Pgp inhibitors; they were more potent and displayed fewer pharmacokinetic interactions than inhibitors of previous generations but caused toxicity in combination with chemotherapy, which was potentially due to the inhibition of Pgp expressed in normal tissue [6]. Furthermore, at higher concentrations, tariquidar and elacridar also inhibit BCRP [183]. Using PET imaging, tariquidar was shown to increase brain levels of Pgp substrates such as verapamil or d-loperamide in rodents, nonhuman primates, and humans, thus demonstrating the utility of transport inhibition at the BBB [6]. However, because of several negative clinical cancer trials with such Pgp inhibitors, interest in investigating these drugs for enhancing drug delivery to the brain has waned.

In the present review, we discuss specific strategies for interfering with Pgp activity at the BBB. The example of seizure-induced overactivity of Pgp at the BBB discussed in Section 2.3 illustrates that interfering with the signaling pathway leading to Pgp induction is much more selective and tissue-specific than the mere direct inhibition of Pgp throughout the body. Some of the drugs involved in this pathway, e.g., COX-2 inhibitors, are clinically available and have been shown to increase the penetration of Pgp substrates into the epileptic brain. However, most of the other novel strategies discussed here are only based on in vitro findings and need to be confirmed in in vivo models for subsequent translation to the clinic, as discussed in Section 5. Nevertheless, continuing to uncover the regulation of Pgp at the BBB has the potential to radically change the way that we facilitate drug penetration across the BBB in the treatment of brain diseases.
